# Oocyte Cryopreservation for Medical and Planned Indications: A Practical Guide and Overview

**DOI:** 10.3390/jcm12103542

**Published:** 2023-05-18

**Authors:** Eric Han, David B. Seifer

**Affiliations:** Department of Obstetrics, Gynecology & Reproductive Sciences, Yale School of Medicine, New Haven, CT 06510, USA; david.seifer@yale.edu

**Keywords:** oncofertility, oocyte cryopreservation, egg freezing, planned oocyte cryopreservation, elective oocyte cryopreservation, medically indicated oocyte cryopreservation, vitrification, fertility preservation

## Abstract

Oocyte cryopreservation (OC) is the process in which ovarian follicles are stimulated, the follicular fluid is retrieved, and mature oocytes are isolated and vitrified. Since the first successful pregnancy utilizing previously cryopreserved oocytes in 1986, OC has become increasingly utilized as an option for future biologic children in patients facing gonadotoxic therapies, such as for the treatment of cancer. Planned OC, also termed elective OC, is growing in popularity as a means to circumvent age-related fertility decline. In this narrative review, we describe both medically indicated and planned OC, focusing on the physiology of ovarian follicular loss, OC technique and risks, timing of when OC should be performed, associated financial considerations, and outcomes.

## 1. Introduction

It has been over 40 years since the first baby was born using in vitro fertilization (IVF). Since then, the field of assisted reproductive technology (ART) has greatly expanded, with over 8 million babies born worldwide through the use of IVF [1]. According to the most recent complete data from the Society for Assisted Reproductive Technology (SART) National Summary Report, there were nearly 300,000 ART cycles in the United States in 2019, of which nearly 16,000 were for oocyte cryopreservation (OC) [2]. Since the first successful pregnancy utilizing cryopreserved oocytes occurred in 1986 [3], there has been significant advancement in laboratory techniques to allow for cryopreserved oocytes to be a viable option for future fertility in both medical (i.e., cancer) and planned (i.e., delayed child bearing) indications [4].

Traditionally, OC has been reserved for patients facing gonadotoxic therapy for the treatment of cancer, such as chemotherapy or pelvic irradiation and, in 2013, the American Society for Reproductive Medicine (ASRM) removed the experimental label on OC for such patients [5]. Nearly 200,000 reproductive-aged individuals are diagnosed with cancer every year in the United States [6]. Presuming an equal ratio of males to females, there is clearly a large need for access to OC. While the use of OC in patients facing gonadotoxic therapies is largely accepted, it was not until 2018 that ASRM concluded that planned OC, more colloquially known as elective or social egg freezing, was ethically permissible [7]. Advertisements for egg freezing are increasingly common, with more women reporting awareness about the procedure from the media rather than a medical professional [8]. Accordingly, some fertility clinics have shifted to specifically market this service. With growing exposure and public interest, providers will inevitably encounter questions from patients on this option. Yet, knowledge on OC across various specialties, including within obstetrics/gynecology (OB/GYN), is limited [9,10]. In this narrative review, we aim to provide a comprehensive overview of both medically indicated and planned OC for providers inside and outside of reproductive medicine.

## 2. High Demand and Need for Increased Utilization

Of the 200,000 reproductive-aged women and men diagnosed with cancer each year in the United States, an estimated 16,000 cases will occur in young children and adolescents [6,11]. Fortunately, death rates for childhood and adolescent cancers have steadily declined by 2.1% per year since 1975, with overall 5-year relative survival rates of 86% [11,12]. As of 2010, there were nearly 380,000 survivors of childhood and adolescent cancer, 70% of whom are over the age of 20 [12]. With continued improvement in cancer mortality, there has been an increasing focus on improving care and quality of life in the context of long-term survivorship [13].

Concern over future fertility is common among cancer patients. Over half of patients newly diagnosed with cancer express a desire for children in the future and 13–16% report an increase in their desires to have children after diagnosis [14,15]. Fertility concerns are also common among parents of childhood cancer survivors but are often overshadowed in the immediate aftermath of a cancer diagnosis [16,17,18]. Both the ASRM and American Society for Clinical Oncology (ASCO) recommend early fertility preservation (FP) counseling and referral to a reproductive endocrinologist and infertility (REI) specialist for patients facing potentially gonadotoxic therapies [19,20]. Yet, few women of reproductive age diagnosed with cancer receive fertility counseling and a smaller proportion (1–2%) ultimately go through any type of FP, including IVF, OC, embryo cryopreservation with or without donor sperm, ovarian transposition, or ovarian tissue cryopreservation [21,22]. Adult women ≤ 35 years of age are more likely to undergo FP procedures but still at a low rate of 6.3% [22]. Inequitable access to FP counseling and treatment has also been linked to socioeconomic status, education level, insurance coverage, and race/ethnicity [23,24,25,26].

There has been an 880% increase in OC cycles in the United States between 2010 and 2016, and it is difficult to delineate between those performed for elective versus medical indications [27]. More recent SART data show a less dramatic but continued increase in OC cycles between 2016 and 2019 of 89% [2]. However, it is reasonable to attribute some of this to the increased utilization of planned OC. Supporting this notion is the decreasing average age of those undergoing OC for any indication, from 36.7 years in 2010 to 34.7 years in 2016 [27].

Studies of individuals pursuing graduate education and medical professionals have consistently shown that time-sensitive fertility knowledge is lacking, natural fertility is over-estimated, and there is lack of alignment between women’s professional and family-building goals [8,9,28,29,30]. In addition, lack of a currently suitable partner and financial concerns are other commonly cited reasons to delay childbearing [8,29,31]. The ethical arguments for and against wide utilization of planned OC center around patient autonomy versus non-maleficence and justice [7]. Planned OC allows for increased flexibility with life circumstances, increasing options to have genetically related offspring at a time when natural fertility would be in decline. On the other hand, concerns over the inherent risks of single and multiple ovarian stimulation(s) and retrieval(s), absence of long-term outcomes, and inequitable access have been raised [7,32].

## 3. Ovarian Follicular Loss: Iatrogenic and Age-Related

The treatment of cancers with chemotherapy, radiotherapy, and/or surgery is a well-established iatrogenic cause of ovarian damage. Table 1 lists various oncologic treatment regimens and their relative gonadotoxicity risk. Total body and pelvic radiotherapy, conditioning chemotherapy regimens for bone marrow transplantation, and alkylating chemotherapy agents are particularly gonadotoxic, with high rates of post-treatment premature ovarian insufficiency (POI) and infertility [33,34]. Treatments assessed to have high gonadotoxicity have >80% likelihood of causing permanent amenorrhea, whereas intermediate gonadotoxicity is associated with 60–80% risk [35]. Those with low gonadotoxicity risk have favorable post-treatment rates of return of spontaneous menses and fertility, though menopause may occur earlier [35,36]. Anti-metabolites (e.g., 5-fluorouracil, 6-mercaptopurine, and methotrexate), vinca alkaloids (e.g., vinblastine and vincristine), anthracyclines (e.g., doxorubicin and daunorubicin), and topoisomerase inhibitors (e.g., topotecan and etoposide) are thought to have low gonadotoxicity [33,34]. Various mechanisms of chemotherapy-related ovarian follicle loss have been suggested, including failure of double-stranded DNA break repair; induction of follicular growth and apoptosis via activation of the PI3K/PTEN/Akt pathway, with compensatory activation of primordial follicles; and damage to the ovarian stroma and microvasculature [33,37].

Gonadotoxicity from radiotherapy results from direct DNA damage and is dependent on the irradiated field and dose administered. The human oocyte is particularly sensitive to radiation, with developing (active) follicles being more radiosensitive than primordial (dormant) follicles [33,38]. Still, the dose of radiation to destroy 50% of primordial ovarian follicles has been estimated to be as low as <2 Gy [39,40]. Furthermore, the effective sterilizing dose, at which fewer than 1000 primordial follicles are expected to survive (akin to the level remaining at menopause), is inversely related to patient age. For a 10-year-old, the effective sterilizing dose is estimated to be 18.4 Gy, whereas it is around 11.5 Gy for a 40-year-old [40]. Total body irradiation as preparation for hematopoietic stem cell transplantation generally involves a total dose of 12–15 Gy in fractionated doses [41]. Brachytherapy for cervical cancer can involve substantially higher doses, exceeding 80 Gy [42]. In addition to direct apoptotic effects on ovarian follicles, a deleterious effect on fertility can result from hypothalamic–pituitary–ovarian (HPO) axis disruption from cranial irradiation and uterine damage [38]. Radiation can induce damage to the uterine myometrium, endometrium, and vasculature, resulting in fibrosis, stunted growth potential, and downstream negative pregnancy outcomes [43]. Surgery that necessitates removal of the ovaries or other organs of the female reproductive tract can clearly impact fertility and the ability to carry a pregnancy.

Finally, it is worth noting that any number of benign conditions and non-oncologic therapies have the potential to impact fertility. Ovarian surgery for benign gynecologic conditions, such as endometriomas, can dramatically reduce ovarian reserve [44]. The treatment of various autoimmune conditions, such as systemic lupus erythematosus, frequently involves gonadotoxic medications [45]. Certain genetic conditions are associated with accelerated follicular loss and risk of POI, such as FMR1 premutation carriers, Turner syndrome, and galactosemia. The limited data on fertility preservation, specifically OC, in these patient populations suggest blunted responses to ovarian stimulation and higher rates of oocyte aneuploidy [46]. As such, early diagnosis and consideration of OC prior to the onset of POI is paramount.

The reproductive timeframe (i.e., biological clock) is relatively narrow in females compared with males, owing to the progressive loss of ovarian follicles with age. The peak number of follicles exists in fetal life during the second trimester, with approximately 6–7 million primordial follicles [47]. From that point, there is a progressively accelerating rate of loss, declining to 1 million at birth to 25,000 at age 37 to 1000 by age 51 (average age of menopause) [48,49]. Moreover, there is an increasing rate of aneuploidy in conjunction with this shrinking follicular pool. This combination of time-sensitive effects results in an age-related decline in fertility such that the relative fertility rate of someone in their early 30s is 15–19% lower than that of someone in their early 20s, 26–46% lower by the late 30s, and a striking 95% lower by the early 40s [50]. In contrast, age affects male fertility in a much more blunted fashion. Spermatogenesis continues well into the later years of life and, while semen parameters decline after age 35, there is not an appreciable decrease in fertility until the late 40s and early 50s, and it is accompanied by a concomitant rise in mutations within sperm [50].

Ovarian reserve is clinically estimated using serum antimullerian hormone (AMH) and early follicular follicle stimulating hormone (FSH) with estradiol (E2), and ultrasonographically with an early follicular antral follicle count (AFC). AMH is a glycoprotein in the transforming growth factor-β family and is a proxy for the functional ovarian pool that is currently available [51]. AMH levels rise in adolescence, peak by the mid-20s, and then progressively lower to negligible levels by menopause [52]. AMH is relatively stable in value across and between menstrual cycles and is best utilized to predict the response to stimulation during IVF [53]. Despite the temptation to use AMH as a fertility marker, it has not been shown to predict fecundability in a non-infertile population and should not routinely evaluated in this group [54]. It may, however, provide useful information for prospective reproductive planning in patients who risk early loss of fertility and may be monitored to assess for the likelihood of reproductive capacity after gonadotoxic treatment [55,56]. Certain types of cancer may reduce AMH levels. Patients with lymphoma have been shown to have lower AMH levels compared with healthy age-matched controls as well as those with other malignancies (e.g., breast, cervical, colon, endometrial, brain, and leukemia), possibly owing to elevated cytokine levels [57,58]. Combined oral contraceptive pills (containing both estrogen and progesterone) are known to temporarily lower AMH levels by 19–30% and should be discontinued 2–3 months prior to testing [59,60,61]. Other hormonal contraceptive methods, such as progestin intrauterine devices (IUDs), subcutaneous implants, vaginal rings, and progestin only pills, can also negatively affect AMH levels; non-hormonal IUDs (e.g., copper IUD) do not impact AMH levels [61].

## 4. Oocyte Cryopreservation Technique

The process of folliculogenesis is a continuous, random process and progresses through the primordial, primary, secondary, preantral, and antral stages. Follicular growth up until the preantral/antral stages is gonadotropin-independent, beyond which it is dependent on follicle-stimulating hormone (FSH) and luteinizing hormone (LH) [62]. In a normal menstrual cycle, around 10 antral follicles start with gonadotropin-dependent growth, but gradual lowering of FSH levels during the follicular phase limits the time it is above a critical threshold, ultimately favoring mono-follicular growth [63]. Typically, only the one follicle with the highest sensitivity to FSH will continue to be stimulated and ovulate [62]. Primordial follicles are arrested in prophase I (termed a germinal vesicle (GV) oocyte) and meiotic competence is not gained until just prior to ovulation [64]. Following the LH surge, the oocyte progresses to metaphase of meiosis II and becomes arrested at this stage; completion of meiosis does not occur until fertilization.

**Figure 1:**
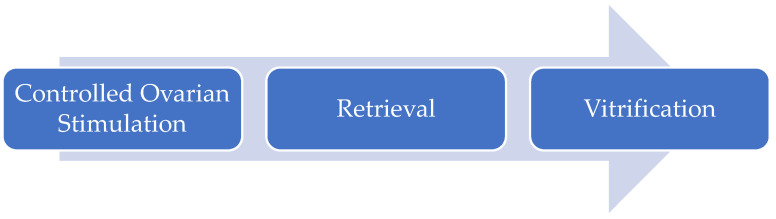
General process of oocyte cryopreservation.

Regardless of the indication, the general process of oocyte cryopreservation is relatively straightforward and involves a few key steps (as outlined in Figure 1): controlled ovarian stimulation (COS) → oocyte retrieval → cryopreservation of mature oocytes (as only mature oocytes are capable of undergoing fertilization). Administration of exogenous gonadotropins during COS with daily injections effectively extends the timeframe of the aforementioned FSH threshold, allowing for multi-follicular development. There are various ovulation induction regimens/protocols to achieve ovarian stimulation and a more in-depth review on COS is beyond the scope of this review. Typically, patients will require 7–12 days of ovarian stimulation, during which their progress will be monitoring on a periodic basis using transvaginal ultrasonography and serum hormone levels. Typically, once 1–2 follicles are measured to be >18 mm, follicle maturation is “triggered” using medications that mimic the natural LH surge. The oocyte retrieval is performed around 36 h after the trigger medication to maximize oocyte maturation rates but minimize the risk of spontaneous ovulation [65]. The oocyte retrieval is an outpatient procedure of less than 30 min during which needle aspiration of the ovarian follicle contents is performed under transvaginal ultrasonography guidance. It is within this fluid that oocytes are isolated and selected for cryopreservation by the embryologist. The entire process can generally be accomplished in two weeks. In patients who are able to delay gonadotoxic treatment for a longer duration of around 4 weeks, a DuoStim protocol, in which a second OC cycle is initiated shortly after the first retrieval, can increase the number of mature eggs frozen [66,67].

Complications related to ovarian stimulation include ovarian hyperstimulation syndrome (OHSS), adnexal torsion, and thromboembolism. Ovarian hyperstimulation syndrome is characterized by internal fluid shifts from the intravascular to extravascular spaces due to increases in vascular permeability. Human chorionic gonadotropin (hCG), commonly utilized as the trigger medication, is thought to have a central role in the pathogenesis by inducing the release of vasoactive substances, particularly vascular endothelial growth factor (VEGF) [68,69]. Clinical features range from abdominal distension, mild nausea, and diarrhea in mild cases and can progress to severe/critical stages complicated by oliguria, severe ascites, hemoconcentration, thromboembolism, arrhythmias, pleural effusions, adult respiratory distress syndrome, and/or sepsis [68]. Features of mild OHSS can be present in 20% of IVF cycles but moderate and severe forms are much less common at <5% [69,70,71]. Mortality is exceedingly rare. Anticipation of a high risk of OHSS is associated with a high AMH (>3.3 ng/mL), in which case certain precautions may be taken, such as pretreatment with metformin, cabergoline at the time of trigger, and use of a gonadotropin-releasing hormone (GnRH) agonist instead of hCG for the final maturation of the pre-ovulatory follicles [72]. Other risk factors for OHSS include early follicular phase AFC > 8, estradiol levels > 3500 pg/mL during COS, polycystic ovary syndrome, a low body mass index (BMI), and high numbers of oocytes retrieved (≥24) [68,72]. Early recognition and management by an experienced REI specialist are critical to mitigating the sequalae of OHSS. Avoiding severe OHSS is particularly relevant in patients undergoing medically indicated OC as it may delay oncologic treatment for several weeks until it resolves. Risks of adnexal torsion and thromboembolism are both increased with OHSS, but remain low overall at <0.2% [69].

Oocyte retrieval is an overwhelmingly safe procedure, with complications such as major bleeding, infection or abscess, and injury to surrounding structures estimated to occur in fewer than 1% of cases [70,73]. Data from nearly 24,000 consecutive oocyte retrievals performed at a single center over a 10-year period noted an overall complication rate of just 0.76%, with hemoperitoneum as the most common (0.23%), followed by pelvic pain and anesthesia complications (both 0.06%), infections (0.04%), and vaginal wall bleeding (0.01%) [73].

Owing to their high water content, human oocytes are particularly susceptible to damage from the freezing and thawing process. Initially, slow freezing was utilized but was plagued by low survival and pregnancy rates [74]. However, the introduction and continued refinement of vitrification (rapid cooling to −196 °C) that avoids the formation of damaging ice crystals has dramatically improved oocyte survival and allowed for pregnancy and live birth outcomes similar to those achieved from freshly retrieved oocytes [74,75,76]. Indeed, it is the vitrification process that has enabled oocyte cryopreservation to be a viable option for fertility preservation.

While the thaw survival rates of mature metaphase-II (MII), immature metaphase-I (MI), and immature GV oocytes are comparable, generally only MII oocytes are cryopreserved owing to the reduced reproductive capacity of immature oocytes that must undergo in vitro maturation (IVM) post vitrification and warming prior to fertilization by intracytoplasmic sperm injection (ICSI) [77,78,79]. Fasano et al. (2012) compared IVM rates of MI and GV pre- and post-vitrification and found higher rates of maturation in those that underwent IVM prior to vitrification rather than after (46% vs. 23.8%). Similar results were seen by Cao et al. (2009). Oocytes that underwent IVM prior to vitrification had higher maturation rates (70.4% vs. 50.8%). No differences in fertilization or cleavage stage embryo development were observed [77,78]. Accordingly, MI-oocytes are often allowed the opportunity to mature to MII-oocytes in culture. While these delayed MI–MII-oocytes have lower fertilization, blastocyst formation, and euploidy rates compared with those that are MII at the time of retrieval, the resulting pregnancies have similar live birth rates [80].

## 5. Timing of Oocyte Cryopreservation

As noted above, the process of OC can generally be completed in two weeks and should be considered if such a delay in initiating oncologic treatment is medically appropriate. Research in breast cancer patients has found mixed data on whether pursuing FP delays initial chemotherapy. At most, the delay is minimal and does not seem to impact invasive-disease-free or overall survival rates [81,82,83]. If unable to take place before initiation of gonadotoxic therapy, OC should be pursued later during a prolonged treatment-free period owing to concerns over the diminished response to ovarian stimulation and teratogenic effects [84]. Animal studies have shown increased miscarriage, aneuploidy, and fetal malformation rates in pregnancies resulting from oocytes being exposed to chemotherapy, with decreasing risk as the time between exposure and ovulation increases [85,86]. Reassuringly, large population-based human studies have not shown increased chromosomal abnormalities in the children of patients who were previously treated with radiation or chemotherapy [87,88]. Still, many advocate to wait at least 6 months (the length of follicular development) from the completion of chemotherapy and/or radiotherapy prior to conception attempts or oocyte/embryo cryopreservation owing to possible teratogenic effects and increased obstetric complications [84,85,89,90]. Uterine compromise from previous radiotherapy increases the risk of miscarriage, preterm delivery, intrauterine growth restriction, and low birth weight [84,89,90]. In contrast, adverse obstetric/perinatal complications are not consistently observed after chemotherapy, particularly beyond 6 months post-treatment [84,89,91,92].

Given that oocyte quantity and quality are inversely related to age, pursuing planned OC at or prior to onset of natural fertility decline is advised. Doyle et al. (2016) analyzed 128 autologous IVF cycles from a pool of 1171 OC cycles, including 1283 previously vitrified and warmed oocytes, and determined age 38 to be the cutoff at which clinical pregnancy rates are worse (60.2% for <38 years vs. 43.9% ≥ 38 years). Other studies have suggested similar age thresholds (between 35 and 38 years) for improved outcomes, including oocyte survival and cumulative live birth rate [93,94,95]. The European Society of Human Reproduction and Embryology (ESHRE) Task Force on Ethics and Law recommends planned OC to be performed before age 35, the upper age limit typically used by oocyte donor programs, and should not be recommended after age 38 [96]. However, this document was released in 2012 and is not in keeping with current practices, as the mean age at which individuals pursue planned OC is beyond age 35 and closer to age 37–38 [31,97]. Indeed, ASRM acknowledges that the available data support improved outcomes for women who undergo planned OC at a younger age but there are insufficient data to pinpoint an optimal age [98].

A novel approach to counsel on the ideal age for planned OC focuses instead on cost effectiveness. Devine et al. (2015) conducted a cost-effectiveness analysis comparing three strategies: (1) planned OC at age 35 with utilization after 6 months of unsuccessful attempts at natural conception upon turning 40; (2) planned OC at age 35, attempting spontaneous conception at age 40 and proceeding with two fresh IVF cycles if unsuccessful; and (3) no planned OC at age 35 and proceeding with two fresh IVF cycles after 6 months of unsuccessful attempts at natural conception upon turning 40. The first strategy (planned OC at age 35 and utilization at age 40) was found to be the most cost-effective, with a 62% predicted live birth rate (LBR) at a cost of $39,946. Strategy 2 yielded the highest LBR at 74%, but at a cost of $61,887 per live birth. Strategy 3 had an LBR of 42% at a cost of $55,060 [99]. Furthermore, Strategy 1 remained more cost-effective than Strategy 2 at all ages and was more cost-effective than Strategy 3 until age 38. Therefore, in individuals planning to defer childbearing until age 40, this model supports planned OC up until age 38, after which proceeding directly to IVF is recommended. These findings are largely reinforced by other cost-effectiveness analyses, including a large systematic review and meta-analysis that found that planned OC is cost-efficient at age 35 assuming a utilization rate of 60% and cost-efficient at age 37 if the individual is willing to utilize donor sperm, but deferring OC and proceeding with IVF is favored by age 38 [100,101].

Of note, these models assume a much higher utilization rate (49–60%) of previously cryopreserved oocytes than actual rates, which range between 7.4% and 38% [93,102,103]. At least one study suggests a lower utilization rate for medically indicated OC compared with planned OC at 7.4% vs. 12.5%, respectively [93].

## 6. Utilization by Transmen

Counseling on fertility preservation prior to gender-affirming treatment is recommended by the ASRM, the World Professional Association of Transgender Health (WPATH), and the Endocrine Society [104,105,106]. While the majority of transmen report a desire to parent a child, far less ultimately pursue OC, citing cost, unwillingness to postpone gender-affirming treatment, distress with the process, fear of gender dysphoria from hormonal treatment, and concern over attitudes from medical staff as barriers to treatment [107,108]. Furthermore, it is well established that fertility preservation treatment is much more common in transwomen than in transmen, with one systematic review noting a utilization rate of 9.6–81.8% compared with just 0–16.7% [107,108,109]. Rather than a difference in the underlying desire to have children, this discrepancy is likely attributed more to the higher barriers inherent to the OC process, including increased cost, invasiveness of an oocyte retrieval, and the need to be managed by a fertility specialist [108]. History of testosterone treatment is not a contraindication to OC and the data are reassuring in terms of outcomes [110,111]. Typically, testosterone treatment is held for several months prior to controlled ovarian stimulation. However, case reports suggest patients can remain on even high-dose testosterone therapy without a deleterious effect on cycle outcome [112,113,114]. This may be an attractive option for those wishing to avoid stopping gender-affirming hormonal treatment and minimize feelings of gender dysphoria.

## 7. Financial Considerations

In the United States, few states have mandated OC benefits for patients facing gonadotoxic therapies and pursuing OC is often cost-prohibitive for many patients. A cycle of oocyte or embryo cryopreservation can be expected to cost around $12,000 USD [115]. In contrast, sperm cryopreservation is considerably less costly, at several hundreds of dollars per collection. Yearly storage fees continue to add to the financial burden. It is of no surprise that, compared with countries where fertility preservation (FP) treatment prior to gonadotoxic therapy is covered (e.g., Israel, France, and Spain), U.S. women report significant funding concerns and guilt over accruing additional debt [116,117,118]. Women who pursued FP prior to cancer therapy are 1.5 times as likely to report financial hardship than those who did not [21]. Of those who ultimately proceed with OC, 50% required additional financial assistance from family members, fundraising, or loans [117].

In January 2018, Connecticut and Rhode Island were the first states to mandate coverage for FP in patients facing medically necessary but potentially gonadotoxic therapies. Connecticut House Bill No. 7124 was championed by then state representative Matthew Lesser and Melissa Thompson, both cancer patients. Passed in June 2017 and effective as of January 2018, the bill was essentially a rewording of an existing mandate so that fertility services would be covered when medically necessary, including prior to cancer treatment. As of January 2023, there are 12 total states with similar mandates and a further 12 with active legislation [119]. It is important to note these mandates only include patients covered under Medicaid in two states, Illinois and Utah [120]. Continued expansion is encouraging as states with comprehensive insurance mandates result in greater utilization of services and safer ART practices [121].

For planned OC, insurance coverage in the United States is the exception rather than the norm. However, expansion of fertility coverage by large employers such as Apple, Google, Netflix, Starbucks, Spotify, and Facebook is steadily increasing as a mechanism to recruit and retain employees [122]. According to the Mercer National Survey of Employer-Sponsored Health Plans, 19% of employers with 20,000 or more employees offered planned OC benefits in 2020 compared with 6% in 2015 [123]. This is still far behind IVF coverage, however, which increased from 36% to 42% between 2015 and 2020. For companies with 500 or more employees, only 11% offered coverage for planned OC in 2020 [123]. Studies have shown that more comprehensive insurance coverage increases patient willingness to consider planned OC and results in increased utilization [8,124]. Cardozo et al. (2020) found that 81% of surveyed graduate students would be more likely to consider planned OC if it were covered by insurance or paid for by their employer. In addition, a survey of medical students found that 73% would consider planned OC if it were covered [28].

Some have raised concern that offering coverage for planned OC may unintentionally coerce individuals to pursue OC to demonstrate commitment to their career [125]. While many women report conflict between their family-building goals and career ambitions, women who would not or were undecided on their decision to pursue planned OC did not consider employer coverage to be coercive [28]. Furthermore, the majority would not change their time frame for having children depending on the presence of financial coverage for OC [8,28].

Private foundations can offer grants in the form of financial assistance or donated infertility services. A survey of 20 such foundations found that the average grant was valued at $8191, ranging from $500 to $25,000 [126]. The majority were provided by a single foundation to patients with a history of cancer. Many (12/20) foundations offered assistance for medically indicated OC, but only five also included planned OC.

## 8. How Many Is Enough?

Perhaps one of the most logical and consequential questions raised by any patient undergoing OC relates to how many eggs are enough. As noted earlier, ovarian aging is a consequence of declining quantity and quality; with age, there is an increasing rate of aneuploidy in a diminishing pool of ovarian follicles. Therefore, the age of the patient at the time they pursue OC has a strong influence on the number needed for a reasonable chance of live birth. Furthermore, it is anticipated that fewer oocytes are retrieved with increasing age. In a study of 3362 patients undergoing their first ovarian stimulation cycle, the median number of oocytes retrieved was greatest in the <30 year group at 18 (interquartile range (IQR) 11–24) and decreased linearly to 8 (IQR 4–12) in the ≥44 year group [127]. In general, it is preferable to freeze as many mature oocytes as possible, as the estimated efficiency from a vitrified and warmed oocyte to a live-born child is only 6.5% per oocyte, ranging from 5.2% for women aged ≥38 years to 7.4% for women < 30 years at the time of OC [128]. These rates are comparable to that calculated for fresh oocytes, which is 6.7% overall [117].

Goldman et al. (2017) examined 520 initial ICSI cycles over a 4-year period between 2011 to 2015 to predict the likelihood of achieving one, two, or three live births based on patient age and number of mature oocytes frozen. As the authors intended to formulate a counseling tool specifically for women pursuing planned OC, only those cycles performed for male factor and/or tubal factor infertility were included to better reflect a fertile population. Donor egg cycles were separately examined. Their model assumed a 95% survival rate of thawed mature oocytes for patients < 36 years of age and egg donors and an 85% survival rate for those ≥ 36 years. Furthermore, the age-dependent probability of having a euploid blastocyst and a 60% live birth rate per euploid blastocyst transfer were factored. As anticipated, the model showed that higher numbers of mature oocytes are needed to be frozen with increasing age. For example, an individual ≤ 35 years of age should aim to freeze 10 mature oocytes to have a 70% probability of having at least one live birth. To achieve this same 70% probability, a 38-year-old, 40-year-old, and 42-year-old would require about 20, 35, and 55 mature oocytes, respectively. Table 2 illustrates this relationship assuming 10 frozen oocytes by individuals of differing ages.

A subsequent study by Maslow et al. (2020) examined the likelihood of cryopreserving sufficient oocytes to achieve a 50%, 60%, or 70% estimated live birth rate (eLBR) with one or two cycles of OC. The authors included 1799 planned OC cycles from 1241 non-infertile patients in the analyses and found that two-thirds of patients were able to achieve a 50% eLBR and just over half were able to achieve a 70% eLBR with a single cycle of OC [130]. The data are more reassuring with two cycles of OC, from which nearly 80% reach the 50% eLBR threshold. As expected, there was a significant impact of age, with patients younger than 37.5 years of age significantly more likely to achieve a 60% eLBR with their first OC cycle compared with those older than 37.5. Controlling for age, AMH was also shown to be significantly associated with the probability of eLBR; those with an AMH value greater than 1.995 ng/dL were seven times more likely to achieve a 60% eLBR with the first OC cycle compared with those with an AMH lower than 1.995 ng/dL [130].

## 9. Outcomes

Cobo et al. (2018) compared the characteristics and reproductive outcomes of more than 6000 women who underwent over 8000 medically indicated and planned OC cycles. Patients who underwent planned OC were older, underwent more treatment cycles, had fewer oocytes retrieved per cycle, and had fewer oocytes vitrified per cycle. They were also more likely to return to utilize their vitrified oocytes with a shorter interval between cryopreservation and utilization. The thaw survival rates of vitrified oocytes were similar. The planned OC group had higher implantation rates (42.6% vs. 32.5%), but this did not translate into a difference in clinical or ongoing pregnancy rates. Other smaller studies have shown no differences in the number of vitrified oocytes between medically indicated and planned OC, and the utilization rate remains too low to make meaningful conclusions on differences in reproductive outcomes [97].

Compared with fresh oocytes (i.e., those used immediately for in vitro fertilization), previously vitrified oocytes have similar fertilization and ongoing pregnancy rates [128]. The limited studies on obstetrical and perinatal outcomes from pregnancies resulting from OC are reassuring, with no increase in congenital anomalies compared with naturally conceived pregnancies and no difference in maternal/perinatal complications compared with pregnancies resulting from fresh oocytes [97,131,132].

## 10. Concluding Remarks

As knowledge and social norms continue to evolve, access to OC will increase and become more prevalent. Providers who care for prepubertal or reproductive-aged women need to be aware of OC as a mainstream option to enable having genetically related children for those who face iatrogenic or age-related loss of fertility.

## Figures and Tables

**Table 1:** Gonadotoxicity risk of various oncologic treatments/regimens.

Gonadotoxicity Risk	Treatment/Regimen
High	Conditioning chemotherapy for bone marrow transplantation Total body irradiation Alkylating agents Pelvic radiotherapy Brachytherapy for cervical cancer
Intermediate	Escalated therapy (e.g., BEACOPP) for Hodgkin’s lymphoma Adjuvant chemotherapy agents for breast cancer
Low	Anti-metabolites Vinca alkaloids Anthracyclines Topoisomerase inhibitors

**Table 2:** Probability of at least 1, 2, and 3 live birth(s) with 10 frozen mature oocytes. Adapted from “Predicting the likelihood of live birth for elective oocyte cryopreservation: a counseling tool for physicians and patients.” by R.H. Goldman, et al., 2017, *Hum Reprod*, 32(4), pp. 853–859 [129].

Age in Years	≥1 Live Birth (%)	≥2 Live Births (%)	≥3 Live Births (%)
≤35	69	30	9
38	45	11	2
40	30	5	<1
42	20	2	<1

## Data Availability

Not applicable.
